# A comparative *in vitro* evaluation of self-assembled PTX-PLA and PTX-MPEG-PLA nanoparticles

**DOI:** 10.1186/1556-276X-8-301

**Published:** 2013-06-27

**Authors:** Fei Cui, Yang Li, Shuifan Zhou, Mengmeng Jia, Xiangrui Yang, Fei Yu, Shefang Ye, Zhenqing Hou, Liya Xie

**Affiliations:** 1 Department of Biomaterials, Research Institute for Biomimetics and Soft Matter, College of Materials, Xiamen University, Xiamen 361005, China; 2 Department of Materials Science and Engineering, Research Institute for Biomimetics and Soft Matter, College of Materials, Xiamen University, Xiamen 361005, China; 3 College of Chemistry & Chemical Engineering, Xiamen University, Xiamen 361005, China; 4 The First Affiliated Hospital of Xiamen University, Xiamen 361005, China

**Keywords:** Self-assembly, PLA, Nanoparticles, Paclitaxel, Drug delivery system

## Abstract

We present a dialysis technique to direct the self-assembly of paclitaxel (PTX)-loaded nanoparticles (NPs) using methoxypolyethylene glycol-poly(d,l-lactide) (MPEG-PLA) and PLA, respectively. The composition, morphology, particle size and zeta potential, drug loading content, and drug encapsulation efficiency of both PTX-PLA NPs and PTX-MPEG-PLA NPs were characterized by X-ray diffraction, Fourier transform infrared spectroscopy, transmission electron microscopy, dynamic light scattering, electrophoretic light scattering, and high-performance liquid chromatography. The passive targeting effect and *in vitro* cell viability of the PTX-MPEG-PLA NPs on HeLa cells were demonstrated by comparative cellular uptake and MTT assay of the PTX-PLA NPs. The results showed that the PTX-MPEG-PLA NPs and PTX-PLA NPs presented a hydrodynamic particle size of 179.5 and 441.9 nm, with a polydispersity index of 0.172 and 0.189, a zeta potential of −24.3 and −42.0 mV, drug encapsulation efficiency of 18.3% and 20.0%, and drug-loaded content of 1.83% and 2.00%, respectively. The PTX-MPEG-PLA NPs presented faster release rate with minor initial burst compared to the PTX-PLA NPs. The PTX-MPEG-PLA NPs presented superior cell cytotoxicity and excellent cellular uptake compared to the PTX-PLA NPs. These results suggested that the PTX-MPEG-PLA NPs presented more desirable characteristics for sustained drug delivery compared to PTX-PLA NPs.

## Background

A nano drug delivery system can transport anticancer agents and preferentially reach tumor sites, owing to the advantages of reduced clearance from the reticuloendothelial system (RES), increased tumor accumulation through enhanced permeation and retention (EPR), and effective cellular uptake, as they offer a less invasive alternative compared with conventional therapeutic cocktails (e.g., chemotherapy, radiation therapy, and surgery), thereby minimizing the excessive toxic side effects and maximizing the efficacy of drugs in clinical trials [1,2]. Some characteristics can be the prerequisites for a nano drug delivery system: (1) a low cytotoxicity and the possibility of biodegradability of the vector itself, (2) versatile surface functionalization, (3) a high drug-loaded content related with elevated therapeutic efficacy, (4) a good dispersibility and colloidal stability of the vector in physiological conditions, (5) a low level of protein adsorption related with a prolonged circulation time, (6) a low degree of premature leakage and the possibility for controlled release of drugs, and (7) can be targeted to cell/tissue of choice and effective cellular uptake [3-5].

Self-assembled nanoparticles (NPs) have attracted considerable interest for their potential use in drug delivery and cancer therapy since they can encapsulate a series of poorly water-soluble anticancer drugs and release them in a sustained manner at their target site [6-8]. Self-assembly technique can provide a simple and low-cost method for producing NPs in a controllable way [9]. Polymeric amphiphiles consisting of hydrophilic and hydrophobic parts can form nanosized self-assemblies with a hydrophobic core and a hydrophilic shell. The hydrophilic shell contributes to their prolonged circulation to increase their ability of reaching the target tumor tissue after systemic administration *in vivo*. More importantly, because of their abnormally leaky vasculature and lack of an effective lymphatic drainage system, self-assembled NPs also tend to be accumulated in tumor sites [10].

Paclitaxel (PTX), one of the most exciting anticancer agents, was currently available. It showed effective activity by inhibiting various tumors and had been used clinically in the treatment of metastatic breast cancer, ovarian cancer, and several other malignancies [11]. New research has shown that PTX has antiangiogenic activity by inhibiting vascular endothelial cell proliferation, motility, and cord/tube formation at extremely low concentrations [12]. For these reasons, we chose PTX as the model chemotherapeutic agent. Despite its potent anticancer activity, unfortunately limited by poor water solubility and toxic side effects, it has no great advantage in tumor targeting for drug delivery and cancer therapy [13].

A series of efforts has been directed to the development of alternative delivery systems for PTX. Poly(d,l-lactide) (PLA), a FDA-approved biodegradable and non-cytotoxic material with a good track record in offering great potential for controlled release, has stood out and been extensively used in the formulation of NPs for biotechnology and drug delivery applications [14]. However, in aqueous solution, the drug-loaded PLA NPs presented poor dispersibility and colloidal stability; in addition, the PLA NPs were not amenable to rapid clearance from the circulation by the RES, immediately after their injection into the systemic circulation. A safe and effective way to answer this problem is to design long-circulating NPs with hydrophilic polymers. Polyethylene glycol (PEG), also a FDA-approved polymer highly soluble in water, has been widely used as a long-circulating agent to improve the biocompatibility and increase the colloidal stability of NPs through steric hindrance, which was often incorporated in drug carriers for delivery to the human body, according to its resistance against opsonization, the process through which protein adsorption is enhanced to induce phagocytosis [15-17]. Thereby, methoxypolyethylene glycol-poly(d,l-lactide) (MPEG-PLA) diblock copolymers have been of great interest as a completely biocompatible material for drug delivery [18,19]. Moreover, MPEG-PLA could make long circulation possible for pharmaceutical uses and opened new perspectives for controlled drug delivery in particular.

In this paper, we present a dialysis technique to direct the self-assembly of PTX-loaded NPs using MPEG-PLA diblock copolymers and PLA, respectively. The hydrophobic polymeric core of the platform readily encapsulated the water-insoluble drug for systemic delivery. The physicochemical properties of the PTX-MPEG-PLA NPs were characterized by Fourier transform infrared spectroscopy (FTIR), X-ray diffraction (XRD), dynamic light scattering (DLS), static light scattering (SLS), transmission electron microscopy (TEM), and confocal laser scanning microscopy (CLSM). *In vitro* drug release profiles and cytotoxicity tests were also conducted. The PTX-PLA NPs were also prepared and characterized in the same way and used for comparison.

## Methods

### Materials

PTX (purity grade > 90%) was purchased from Qilu Pharmaceutical Co., Ltd. (Shandong, China). PLA (50 kDa) and MPEG-PLA (10%) were provided by Daigang BIO Engineer Co., Ltd. (Shandong, China). A dialysis bag (Mw cutoff = 8,000 to 14,000 Da) was ordered from Greenbird Inc. (Shanghai, China). Double-distilled water was used throughout. All chemical reagents were of analytical grade and used without further purification unless otherwise stated. All solutions used in a high-performance liquid crystal (HPLC, Waters Associates, Milford, MA, USA) analysis were filtered and degassed using a 0.22-μm membrane filter with a filtration system.

### Preparation of the PTX-MPEG-PLA NPs

The PTX-MPEG-PLA NPs were prepared by a facile dialysis method. In brief, 100 mg of MPEG-PLA and 10 mg of PTX were codissolved in 10 mL of organic solvent (acetone, unless specified) accompanied by vigorous stirring; then the resulting organic phase was introduced into a dialysis bag. Subsequently, the dialysis bag was placed with gentle agitation (100 rpm) into 1,000 mL of water as the aqueous phase. The organic phase was dialyzed against the aqueous phase for 6 h. Following this, the aqueous phase was subjected to repeated cycles of replacing with fresh water at designed time points (1, 2, 3, 4, 5, and 6 h) to remove the diffused organic phase by dialysis. The as-prepared PTX-MPEG-PLA NPs were lyophilized for 24 h using a freeze drier (Labconco Plus 12, Labconco, Kansas City, MO, USA) and stored at 4°C for future use. The PTX-PLA NPs were prepared in a similar way by using 100 mg of PLA. The drug loading content and drug encapsulation efficiency of PTX-MPEG-PLA NPs and PTX-PLA NPs were determined by a HPLC system consisting of a Waters 2695 Separation Module and a Waters 2996 Photodiode Array Detector with the following conditions: stationary phase: Thermo C18 column (150 mm × 4 mm, 5 μm), temperature 26 ± 1°C; mobile phase: methanol/ultrapure water (65/35, *v*/*v*), freshly prepared, filtered through a 0.22-μm Millipore (Billerica, MA, USA)membrane filter before use, and degassed utilizing a sonication method; elution flow rate, 0.8 mL/min; and detection wavelength, 227 nm. The concentration of PTX was determined based on the peak area at the retention time of 7.5 min by reference to a calibration curve.

### XRD analysis

The physical state of PTX in the MPEG-PLA NPs or PLA NPs was analyzed using a Philips X’Pert Pro Super X-ray diffractometer (Philips, Amsterdam, Netherlands) equipped with CuKα radiation generated at 30 mA and 40 kV. The diffraction angle was increased from 5° to 60°, with a step size of 0.05. As control, the characteristic of PTX and MPEG-PLA NPs/PLA NPs, and the physical mixture of PTX and MPEG-PLA NPs/PLA NPs with the same ratio were investigated as well.

### FTIR analysis

FTIR spectra were obtained using a NicoletAVTAR36 FTIR spectrometer (Thermo Scientific, Logan, UT, USA) with a resolution of 4 cm^−1^ from 4,000 to 400 cm^−1^. The PTX-MPEG-PLA NPs or PTX-PLA NPs were lyophilized to obtain the FTIR sample. Two milligrams of dried powder was added to 200 mg of KBr. The powder was pressed into a pellet for analysis. Besides, the FTIR spectra of MPEG-PLA NPs/PLA NPs and pure drug were obtained as control.

### Particle size, polydispersity index, surface charge, and morphology

Average particle size and polydispersity index (PDI) were determined by DLS using a Malvern Zetasizer Nano-ZS (Malvern Instruments, Worcestershire, UK). Zeta potential was evaluated by electrophoretic light scattering (ELS) with Zetaplus (Brookhaven Instruments Corporation, Holtsville, NY, USA). Particle size was evaluated by intensity distribution, and particle size distribution was represented by PDI. The morphology of the PTX-MPEG-PLA NPs was observed on a JEM 2100 transmission electron microscope (JEOL, Tokyo, Japan) operating at 200 kV. One drop of the suspension was diluted with water, subsequently placed on a carbon-coated copper grid, and lastly, dried in the air before observation. PTX-PLA NPs were used for comparison.

### *In vitro* drug release behavior

Evaluation of *in vitro* release behavior was conducted to examine how rapidly PTX was released from the PTX-MPEG NPs. The output obtained by the dynamic dialysis method provided a correlation with *in vivo* drug release. The lyophilized NPs (equivalent to 5 mg of PTX) were dispersed in 2 mL of PBS (1/15 M, pH 7.4), and the dispersion was added into a dialysis bag. The release experiment was initiated by placing the end-sealed dialysis bag in 48 mL of PBS (1/15 M, pH 7.4). The system was kept on a magnetic stirrer under controlled conditions (100 rpm, 37°C). At predetermined time intervals, 2 mL of the release medium was completely withdrawn and subsequently replaced with the same volume of fresh PBS solution. The concentration of PTX in the samples was measured by HPLC. The lyophilized PTX-PLA NPs (equivalent to 5 mg of PTX) were used for comparison.

### *In vitro* cellular uptake

*In vitro* cellular uptake was employed to investigate the distribution of PTX-loaded MPEG-PLA NPs in the cell. Following a 24-h culture of HeLa cells in a six-well plate, 100 μL of rhodamine B-labeled PTX-MPEG-PLA NPs (1 mg/mL) was added to the medium and incubated further for 48 h. The HeLa cells were washed five times with PBS and continuously stained with 50 μL of Hochest 33258 (0.005 mg/mL). The cells were observed with CLSM (Leica TCS SP5, Leica Microsystems, Mannheim, Germany). Cells treated with rhodamine B-labeled PTX-PLA NPs were used for comparison.

### *In vitro* cell viability assays

A549 cells were cultured in standard cell media recommended by the American Type Culture Collection. Cells seeded in 96-well plates were incubated with a series of increasing concentrations of PTX-MPEG-PLA NPs for 48 h. Subsequently, relative cell viability was assessed by the standard MTT assay. Cells treated with free PTX and cells treated with the PTX-PLA NPs were compared.

## Results and discussion

### Preparation of the PTX-MPEG-PLA NPs

**Figure 1:**
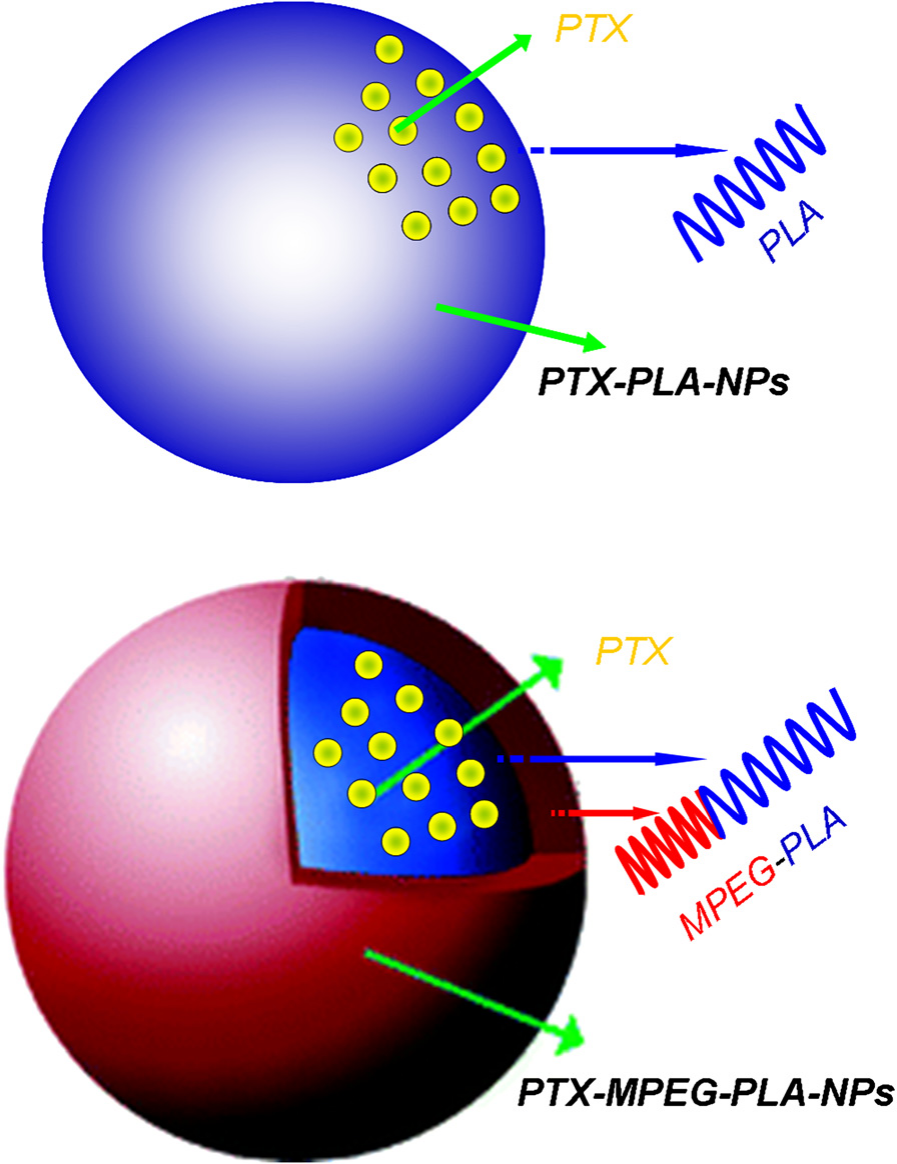
Schematic representations of PTX-PLA NPs and PTX-MPEG-PLA NPs.

Acetone is water-miscible and a good solvent for MPEG-PLA. PTX and MPEG-PLA were first codissolved in this organic phase and was then extensively dialyzed against the aqueous phase. In the dialysis process, acetone was gradually removed and slowly replaced with water, which resulted in the hydrophobic PLA comprising a core while the hydrophilic PEG extended to the outer aqueous environment to form a shell. Thus, a minimal energy state was attained in aqueous media, and the lipophilic drug PTX spontaneously transferred inside the hydrophobic cores of particles because of the driving force of hydrophobic interaction (Figure 1). Furthermore, inter- and/or intramolecular hydrogen bonds between hydroxyl groups of PEG will stabilize the NPs. Thus, the amphiphilic MPEG-PLA can form NPs loading PTX drug with a well-defined core-shell structure by self-assembly in aqueous media, and the structure is believed to possess a self-stabilization function. The determined drug entrapment efficiency and drug-loaded content of PTX-MPEG-PLA NPs by HPLC were 18.3 ± 0.4% and 1.83 ± 0.04%, and those of PTX-PLA NPs were 20.0 ± 0.7% and 2.00 ± 0.07%.

### XRD and FTIR analysis

**Figure 2:**
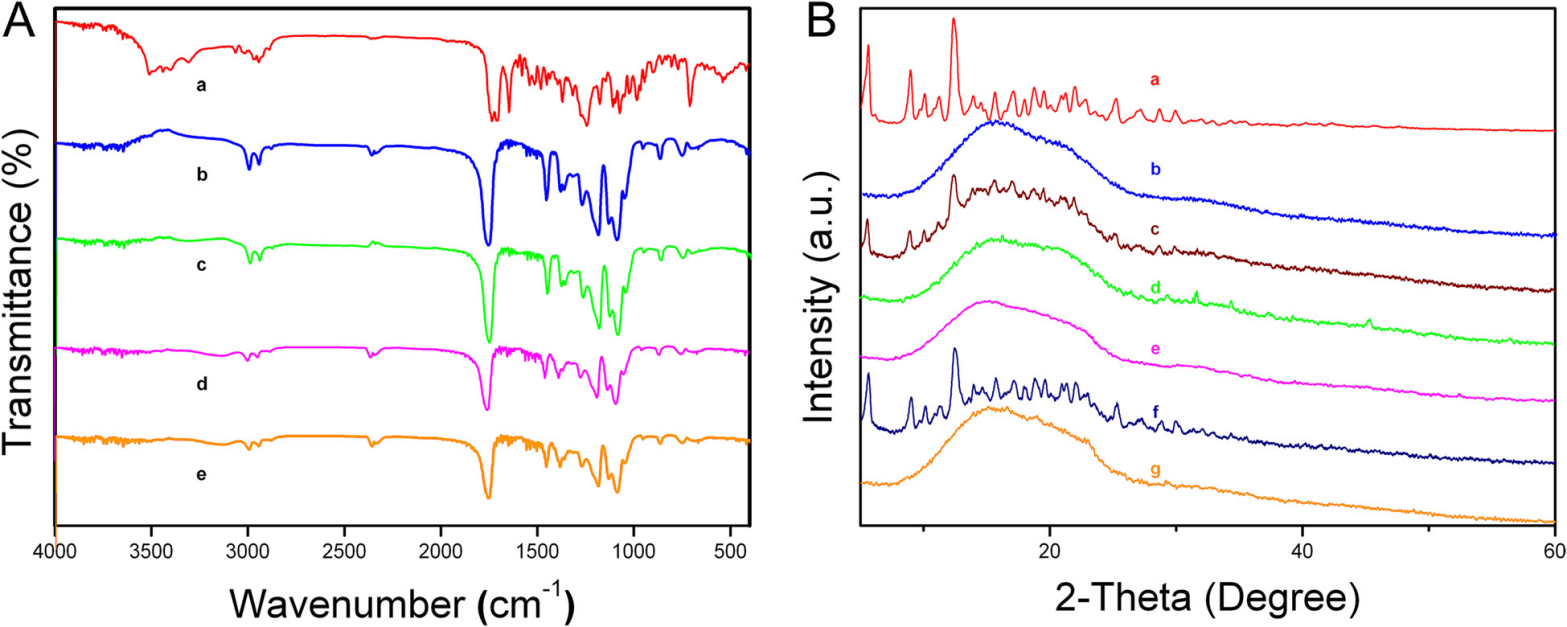
**FTIR and XRD analysis of the PTX, physical mixture, and drug-loaded NPs. (A)** FTIR spectra of PTX (a), PLA NPs (b), PTX-PLA NPs (c), MPEG-PLA NPs (d), and PTX-MPEG-PLA NPs (e). **(B)** XRD patterns of PTX (a), PLA NPs (b), physical mixture of PTX and PLA NPs (c), PTX-PLA NPs (d), MPEG-PLA NPs (e), physical mixture of PTX and MPEG-PLA NPs (f), and PTX-MPEG-PLA NPs (g).

XRD diffraction patterns of PTX, both blank MPEG-PLA NPs and PLA NPs, physical mixture, and drug-loaded NPs are presented in Figure 2B. It was clear that pure PTX showed partially sharp crystalline peaks, representative of the characteristics of a molecular compound with some crystallinity, whereas a broad peak was presented in blank NPs, indicating that blank NPs were amorphous and lack crystalline peaks. Some crystalline drug signals were still detectable in the physical mixture. A decrease in the intensity of the peaks was explained by a lower loading of the drug per unit weight of the physical mixture compared to pure PTX. Conversely, the crystalline peaks almost disappeared in the drug-loaded NPs whereas the amorphous characteristics resembled those of blank NPs, indicating that the drug was encapsulated within the NPs and suggesting that PTX in the NP matrix was molecularly dispersed or in the amorphous form.

The physicochemical state of incorporating drug in the NPs is one important factor that affects the drug release behavior. As shown in Figure 2B, there was no change in the absorption peaks between the blank NPs and drug-loaded NPs. Of note, the absorption peaks of the pure drug were almost shielded because of drug entrapment effect. Based on the spectral characteristics of PTX, blank NPs and drug-loaded NPs, it should be inferred synthetically that there was no chemical interactions between PTX and blank NPs because of no appearances of new functional groups. Therefore, the individual physicochemical characteristics will not change *in vitro* and *in vivo*.

### Particle size, PDI, surface charge, and morphology

**Figure 3:**
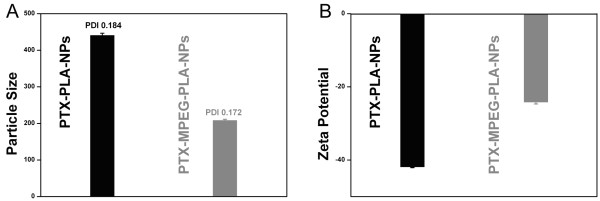
**Particle size and zeta potential.** Particle size determined by DLS **(A)** and zeta potential determined by ELS **(B)** of PTX-MPEG-PLA NPs and PTX-PLA NPs.

DLS and ELS (see Figure 3) showed that the PTX-MPEG-PLA NPs and PTX-PLA NPs presented a hydrodynamic particle size of 179.5 and 441.9 nm, with a PDI of 0.172 and 0.189, and a zeta potential of −24.3 and −42.0 mV, respectively. Smaller particle size favored EPR targeting; lower PDI indicated good dispersibility, a prerequisite of good stability. Higher zeta potential supported that the NPs did not aggregate much in aqueous state in general and in physiologically relevant media in particular. Knowledge on these characteristics of a NP system can help predict the fate and biodistribution of NPs at the cellular or animal level *in vivo* after administration [1,6]. As clearly seen from Figure 3A, the hydrodynamic particle size of PTX-MPEG-PLA NPs was much less than that of PTX-PLA NPs; the particle size is compatible in EPR targeting attributed to the leaky nature of tumor vessels. Therefore, PTX-MPEG-PLA NPs were chosen as an effective model drug carrier as their particle size distribution and zeta potential distribution were narrow.

**Figure 4:**
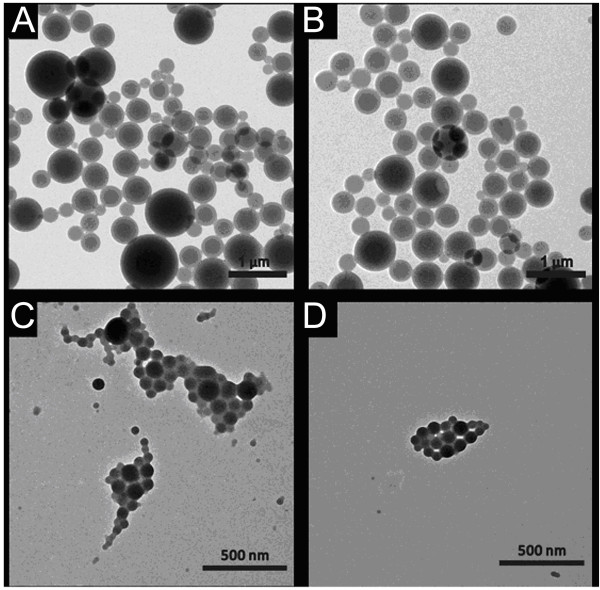
TEM images of PTX-PLA NPs (A, B) and PTX-MPEG-PLA NPs (C, D).

Additionally, TEM images revealed that PTX-MPEG-PLA NPs were regularly spherical in shape and have a generally smooth surface with an approximate average size of around 100 nm, and the core particles contain a lighter outer region (see Figure 4). The average size of these NPs determined by DLS was 179.5 nm, not well consistent with the size determined by TEM. These factors were possibly responsible for the following differences. First, in the case of the TEM method, TEM depicted the size in the dried state of the sample, whereas DLS determined the size in the hydrated state of the sample. Second, the polymer shell of the particle surface tended to expand in aqueous environment which inevitably increased the hydrodynamic size of NPs because of solvent effect. Third, some NPs may be likely aggregated in the aqueous environment.

Dialysis offered an easy and effective method for the preparation of small and well-distributed NPs. At present, the mechanism of NP formation by dialysis method is not fully understood. It was thought that it may be based on a mechanism similar to that of nanoprecipitation. It was based on the utilization of a physical barrier that allowed the passive transport of organic solvents to slow down the mixing of MPEG-PLA with water; the organic solvent played a role in the morphology and particle size distribution of the NPs [20]. The presence of hydrophilic PEG chain, small particle size, high zeta potential, sharp curve of the particle size, and zeta potential distribution indicated that the spherical NPs as effective nano drug delivery systems were expected to be relatively stable in physiologic media for intravenous delivery.

### *In vitro* drug release behavior

The release behavior of the NPs is an important aspect because this information is directly related to the design of nano drug delivery system. Generally, the release of drug from polymeric NPs will depend upon the diffusion rate of the drug from the NPs, NP stability, and the biodegradation rate of the copolymer. If the NPs are stable and the biodegradation rate of the copolymer is slow, the release rate will be most likely influenced by the following factors: the strength of the interactions between the drug and the core block, the physical state of the core, the drug-loaded content, the molecular volume of the drug, the length of the core block, and the localization of the drug within the NPs.

**Figure 5:**
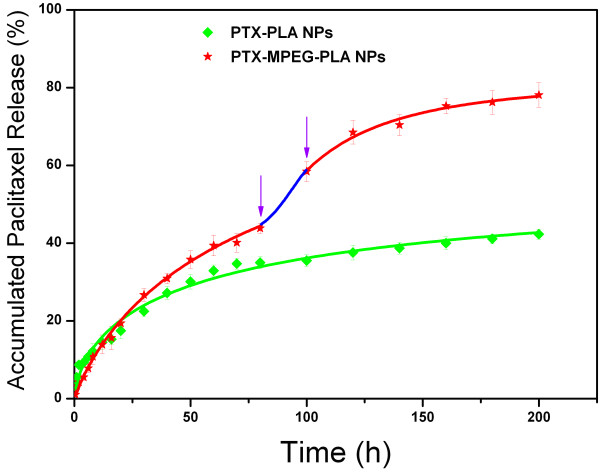
***In vitro *release profiles of PTX-MPEG-PLA NPs versus PTX-PLA NPs in PBS (1/15 M, pH 7.4).** The blue line represents the second phase of burst release. The purple arrows showed their burst start and endpoint.

As shown in Figure 5, PTX-PLA NPs and PTX-MPEG-PLA NPs both presented sustained drug release profiles with about 42.3% and 78.1% of the total PTX released from NPs. The accelerated release may be explained by three factors. First, the particle size of the PTX-MPEG-PLA NPs was much smaller than that of the PTX-PLA NPs, reducing the total releasing time of the drug from the NPs. Second, the presence of hydrophilic PEG in the polymer NPs reduced the hydrophobic interaction between the drug and matrix. Third, the outer PEG molecule could induce easier penetration of the water and facilitated the bulk erosion of the polymer matrix. All the factors, singly or in combination, could promote the release of PTX from the PTX-MPEG-PLA NPs.

Of note, in the case of PTX-PLA NPs, a drug release behavior can be divided into two phases: the first one considered as a relatively fast release phase at the initial stage, commonly ascribing to the easy release of free PTX absorbed on the surface of the NPs by simple diffusion, and subsequently, the second one considered as a constantly prolonged release phase, which is most likely related to the slow transport of drug from the NPs driven by a diffusion-controlled mechanism. In the case of PTX-MPEG-PLA NPs, these release behaviors were different; the first abrupt release of PTX was minor from 0 to 12 h, which may have resulted from the steric effect of long PEG chain, which led to the low risk and reduced toxicity. Subsequently after the long sustained release by a diffusion-controlled mechanism, the second abrupt release of PTX from the NPs presented at 80 h, which was likely attributed to the deprotection of PEG as a result of the hydrolysis of MPEG-PLA, suggesting that the presence of hydrophilic PEG on the surface of NPs could eventually favor PTX to penetrate from the NPs.

### *In vitro* cellular uptake

**Figure 6:**
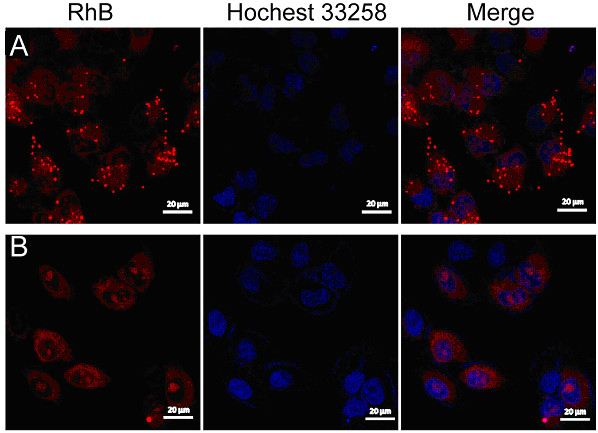
**CLSM images of cells incubated with PTX-loaded NPs which were labeled by rhodamine B.** For each panel, the images from left to right showed rhodamine B fluorescence in cells (red), cell nuclei stained by Hochest 33258 (blue), and overlays of the two images. **(A)** PTX-PLA NPs, **(B)** PTX-MPEG-PLA NPs.

First, as may be seen from Figure 6, a predominant and strong accumulation of red signals in the cell cytoplasm was observed. The phenomenon demonstrated that rhodamine B-labeled PTX-PLA NPs and PTX-MPEG-PLA NPs could be uptaken into the cells. Thus, the NPs entering into cells could play the role of a drug storehouse, effectively exerting their pharmacological and biological effects. Furthermore, it is very interesting to note that the fluorescent signals of PTX-PLA NPs were much stronger than those of PTX-MPEG-PLA NPs. The results were speculated to be associated with these important reasons. Firstly, a great deal of hydrophilic PEG on the surface of MPEG-PLA NPs could prevent the PLA core from transporting across the lipid-rich cell membranes and entering the internal environment of the cells. Secondly, the lipophilicity of PLA facilitated the delivery of NPs to the interior of the cells across the phospholipid bilayer of cellular membranes. Lastly, there is also some contribution of the large particle size of PTX-PLA NPs, which was in favor of entrapping more rhodamine B. In consequence, powerful red fluorescent signals could be seen in the cell. However, there is another possibility that the large particle size of PTX-PLA NPs resulted in the aggregation of NPs. Then the aggregates became too large to enter the cell, so the strong red dot signals were from the PTX-PLA NPs absorbed on the cell surface. In this case, both PTX-PLA NPs and PTX-MPEG-PLA NPs had similar cellular uptake.

### *In vitro* cell viability assays

**Figure 7:**
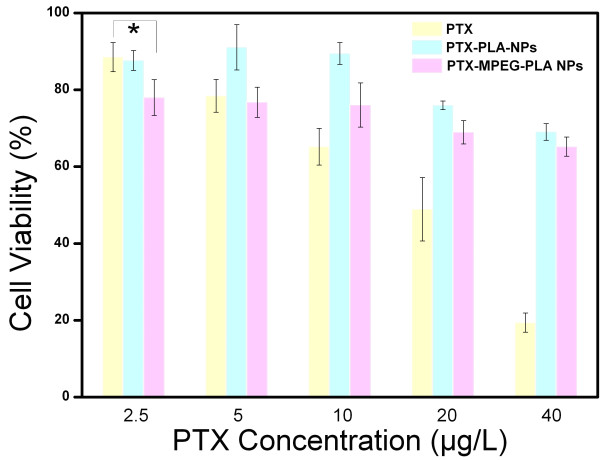
***In vitro *cell viability assays for growth inhibition effect after 48 h (*n*** **= 6).**

As shown in Figure 7, the survival rate of A549 cells was basically suppressed in a drug dose-dependent manner by free PTX, PTX-PLA NPs, and PTX-MPEG-PLA NPs. Interestingly, the lowest concentration group (loaded with an equivalent amount of PTX) of the PTX-MPEG-PLA NPs observably presented lower cell viability than that of free PTX with the concentration of 2.5 μg/mL (*P* < 0.05), indicating that the PTX-MPEG-PLA NPs presented a more effective bioavailability compared with the free PTX solution. On the contrary, the other groups with the concentration of 10, 20, and 40 μg/mL of PTX-MPEG-PLA NPs presented a significantly low level of inhibition effect compared to free PTX. This different phenomenon could be explained by the cell penetration rate of drug depending on NP advantage and drug concentration differences between the internal and external environment of the cell membrane. It should be emphasized that, in the case of the lowest concentration (2.5 μg/mL) of PTX, the NP advantage played a rather important role in the cell penetration rate of drug; their particle size can easily and virtually increase the cellular uptake of drug and the accumulation in the cell through endocytosis mechanism. However, in the case of other high concentrations of PTX (10, 20, and 40 μg/mL), the drug concentration differences played a main role. Most importantly, it was speculated that there were a more sustained release status and depot effect and a longer time to reach release equilibrium for the PTX-MPEG-PLA NPs compared to free PTX. Therefore, the drug was released incompletely from the NPs in 48 h. Thus, PTX-MPEG-PLA NPs are promising in the expansion of dosing range of chemotherapeutic drugs and rendering patients safe cancer therapy. Additionally, it was interesting to note that the cell viability in PTX-MPEG-PLA NPs was higher than that in PTX-PLA NPs at a series of increasing concentrations (2.5, 10, 20, and 40 μg/mL). This result can most likely be attributed to the drug release rate of the PTX-MPEG-PLA NPs being higher than that of the PTX-PLA NPs.

## Conclusions

In our previous study, a simple but successful method was developed to obtain PTX-MPEG-PLA NPs with appropriate formulation characteristics including small particle size, narrow particle size distribution, high zeta potential, satisfactory drug encapsulation efficiency, and appreciable drug-loaded content. The PTX-MPEG-PLA NPs presented a faster drug release rate but minor burst release as well as a higher cell cytotoxicity compared to the PTX-loaded PLA NPs. A further study on the *in vivo* pharmacokinetics and antitumor effects of PTX-MPEG-PLA NPs is currently in progress.

## Competing interests

The authors declare that they have no competing interests.

## Authors’ contributions

FC, YL, and SZ performed the experiments. MJ, XY, FY, and SY were involved in the experimental planning and analysis of the results. ZH and LX designed and planned the experiment and drafted the manuscript. All authors read and approved the final manuscript.
